# Structural Features Promoting Photocatalytic Degradation of Contaminants of Emerging Concern: Insights into Degradation Mechanism Employing QSA/PR Modeling

**DOI:** 10.3390/molecules28062443

**Published:** 2023-03-07

**Authors:** Antonija Tomic, Marin Kovacic, Hrvoje Kusic, Panaghiotis Karamanis, Bakhtiyor Rasulev, Ana Loncaric Bozic

**Affiliations:** 1 Faculty of Chemical Engineering and Technology, University of Zagreb, Marulicev Trg 19, 10000 Zagreb, Croatia; 2 Department for Packaging, Recycling and Environmental Protection, University North, Trg dr. Žarka Dolinara 1, 48000 Koprivnica, Croatia; 3 E2S UPPA, CNRS, IPREM, Université de Pau et des Pays de l’Adour, Hélioparc Pau Pyrénées, 2 Rue de President Angot, 64053 Pau, France; 4 Department of Coatings and Polymeric Materials, North Dakota State University, Fargo, ND 58102, USA

**Keywords:** contaminants of emerging concern, TiO_2_ photocatalysis, degradation, structural influence, QSA/PR modeling

## Abstract

Although heterogeneous photocatalysis has shown promising results in degradation of contaminants of emerging concern (CECs), the mechanistic implications related to structural diversity of chemicals, affecting oxidative (by HO•) or reductive (by O_2_•^−^) degradation pathways are still scarce. In this study, the degradation extents and rates of selected organics in the absence and presence of common scavengers for reactive oxygen species (ROS) generated during photocatalytic treatment were determined. The obtained values were then brought into correlation as *K* coefficients ($M_{\mathrm{HO}•}$/$M_{O_{2}{•}^{-}}$), denoting the ratio of organics degraded by two occurring mechanisms: oxidation and reduction via HO• and O_2_•^−^. The compounds possessing *K* >> 1 favor oxidative degradation over HO•, and vice versa for reductive degradation (i.e., if *K* << 1 compounds undergo reductive reactions driven by O_2_•^−^). Such empirical values were brought into correlation with structural features of CECs, represented by molecular descriptors, employing a quantitative structure activity/property relationship (QSA/PR) modeling. The functional stability and predictive power of the resulting QSA/PR model was confirmed by internal and external cross-validation. The most influential descriptors were found to be the size of the molecule and presence/absence of particular molecular fragments such as C − O and C − Cl bonds; the latter favors HO•-driven reaction, while the former the reductive pathway. The developed QSA/PR models can be considered robust predictive tools for evaluating distribution between degradation mechanisms occurring in photocatalytic treatment.

## 1. Introduction

Contaminants of emerging concern (CECs), such as pharmaceuticals, personal care products, industrial chemicals, per- and polyfluoroalkyl substances (PFASs), and pesticides, have raised strong concerns due to their potential bioaccumulative and toxic characteristics [1,2,3]. Hundreds of thousands of tons of CECs are dispensed and consumed annually worldwide and are continuously discharged into the environment through wastewater treatment plant (WWTP) effluents [4]. Thus, CECs have been detected in various environmental matrices, causing adverse effects such as increased resistance of microorganisms to antibiotics, acute or chronic toxicity, uncertainties related to transformation products and metabolites, and endocrine-disrupting effects [1,5]. Accordingly, the upgrade of WWTPs by advanced treatment technologies as a tertiary step is required to ensure the elimination of health risks posed by CECs in water [5,6]. Advanced oxidation/reduction processes (AO/RPs), which generate highly reactive oxygen species (ROS) (e.g., hydroxyl radicals, HO•; superoxide radicals, O_2_•^−^; perhydroxyl radicals, HO_2_•; hydrogen peroxide, H_2_O_2_; etc.), either in situ or via the activation of added oxidants/reductants/catalysts, have been shown to be effective for elimination of CECs [7,8,9]. Among AO/RPs, heterogeneous photocatalysis, based on well-known reactions (Equations (1)–(3)) initiated by the activation of semiconducting material illuminated by sufficient energy, as follows,

(1) $${\mathrm{TiO}}_{2}\overset{h\nu }{\to }e_{\mathrm{cb}}^{-}\left({\mathrm{TiO}}_{2}\right)+h_{\mathrm{vb}}^{+}\left({\mathrm{TiO}}_{2}\right)$$

(2) $${\mathrm{TiO}}_{2}\left(h_{\mathrm{vb}}^{+}\right)+H_{2}O\to {\mathrm{TiO}}_{2}+\;•OH+H^{+}$$

(3) $${\mathrm{TiO}}_{2}\left(e_{\mathrm{cb}}^{-}\right)+O_{2}\to {\mathrm{TiO}}_{2}+O_{2}{•}^{-}$$

have shown promising effectiveness and suitability for the removal of persistent contaminants [10,11,12]. Titanium dioxide (TiO_2_) appears to be the most extensively studied and used photocatalyst, with still the highest potential for use in the commercial application of photocatalytic treatments [13,14] due to properties such as high stability, cost-effective and favorable performance [10,15]. The effectiveness of photocatalytic treatment, besides key process parameters such as pH and TiO_2_ loading, strongly depends on the structure of organics present. Namely, process parameters such as pH would dictate (i) positive/negative charge of TiO_2_ surface; (ii) present organics in deprotonated or protonated form of undergoing adsorption/degradation; and (iii) susceptibility of compounds to being adsorbed at the photocatalyst surface and be directly degraded there by photogenerated charges (${\mathrm{TiO}}_{2}\left(h_{\mathrm{vb}}^{+}\right)$ and ${\mathrm{TiO}}_{2}\left(e_{\mathrm{cb}}^{-}\right)$). TiO_2_ loading dictates the amount of generated ROS, but also the photo-shielding effect within the reactor space when present in excess [10,15]. On the other hand, the structure of organics would dictate whether degradation undergo preferably oxidation or reduction mechanism. These mechanisms occur simultaneously, but not to the same extent, and consequently, pathways and formed transformation products of organics would differ, affecting the overall quality of treated water in terms of toxicity and biodegradability [16,17,18]. Hence, Chen et al. [19] investigated the kinetics of photocatalytic degradation of aliphatic carboxylic acids by UV/TiO_2_; the results revealed that the degradation mechanism and its efficiency depend on structural features of studied aliphatics, particularly the number of carboxylic groups—more -COOH groups would yield higher degradation rate. However, they did not provide deeper investigation of occurring mechanisms. Furthermore, Yin et al. [20] investigated the degradation of eight aliphatic halogenated contaminants in synthetic drinking water by UVA/TiO_2_ and UVA/Cu-TiO_2_ processes, and established relationships between degradation rate constants and structural characteristics of studied organics by quantitative structure activity/property relationship (QSA/PR) models. Namely, the degradation of contaminants possessing more polar electron withdrawing moieties and higher degrees of chlorination is strongly promoted in UV-A/TiO_2_. Additionally, Huang et al. [21] studied photocatalytic degradation of sulfonamides from the structure-dependent point of view; the findings revealed that degradation rates are strongly related *E*_HOMO_ values, presumably due to the importance of such moiety in HO• attack [22]. It should be noted that these studies were performed on a limited number (≤10 in each study) of organics with rather high similarities within their structures (e.g., aliphatics in the first two studies, sulfonamides in the third study).

There are numerous studies investigating structure-dependent reactivity of organics with HO• derived within various environmental systems/applications [22,23,24,25,26,27]. However, studies directed toward O_2_•^−^ reactivity are rather scarce, particularly related to water treatment systems [20,27]. To the best of our knowledge, studies comprehending the simultaneous involvement of main oxidative and reductive species, HO• and O_2_•^−^, respectively, in photocatalytic systems from the structure–activity aspect have not been performed. Hence, in this study, we investigated the influence of structural features of CECs on their photocatalytic degradation to get insight into the mechanistic aspects facilitating simultaneous indirect oxidation and reduction (i.e., in the bulk). To that end, we have employed QSA/PR modeling to establish the susceptibility of organics based on their molecular structures for oxidative (via HO•) and reductive (O_2_•^−^) degradation by UVA/TiO_2_ process.

## 2. Results and Discussion

### 2.1. Photocatalytic Degradation of Selected Organics

The photocatalytic degradation of organics can occur via one of two mechanisms: (i) direct, occurring at the photocatalyst surface by photogenerated holes (*h*^+^) and electrons (*e*^−^); and (ii) indirect, occurring in the bulk by ROS, primarily HO• and O_2_•^−^, generated as products of reactions of HO^−^ (as water dissociates) and O_2_ (dissolved) with photogenerated *h*^+^ and *e*^−^, respectively (Equations (2) and (3)) [9,10,15]. In this study, we focus only on the indirect degradation by ROS, and in that purpose, experiments with dimethyl sulfoxide (DMSO) and benzoquinone (BQ) as effective scavenging agents for HO• and O_2_•^−^, respectively (Equations (4) and (5)), were performed [16,28].

(4) $$\mathrm{DMSO}+•OH\to \mathrm{products}\;k_{1}=6.6\times 10^{9}\;M^{-1}s^{-1}$$

(5) $$\mathrm{BQ}+O_{2}{•}^{-}\to \mathrm{products}\;k_{2}=5.88\times 10^{10}\;M^{-1}s^{-1}$$

**Figure 1:**
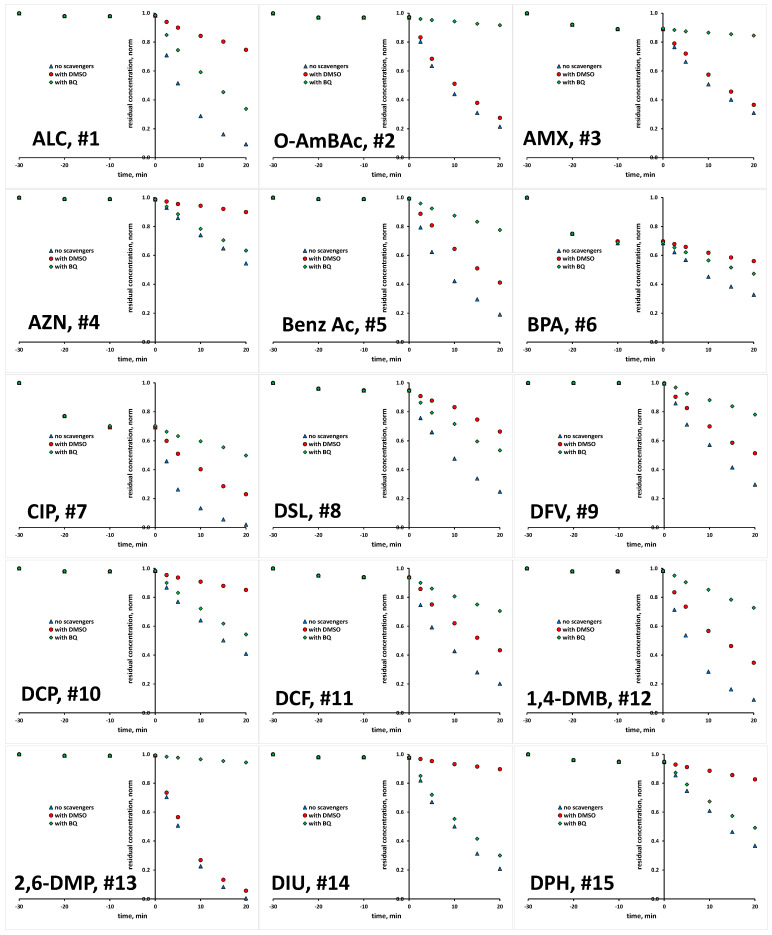
Degradation kinetics of studied organics (#1–15, Table 1) without and with the presence of scavengers for HO• and O_2_•^−^.

**Figure 2:**
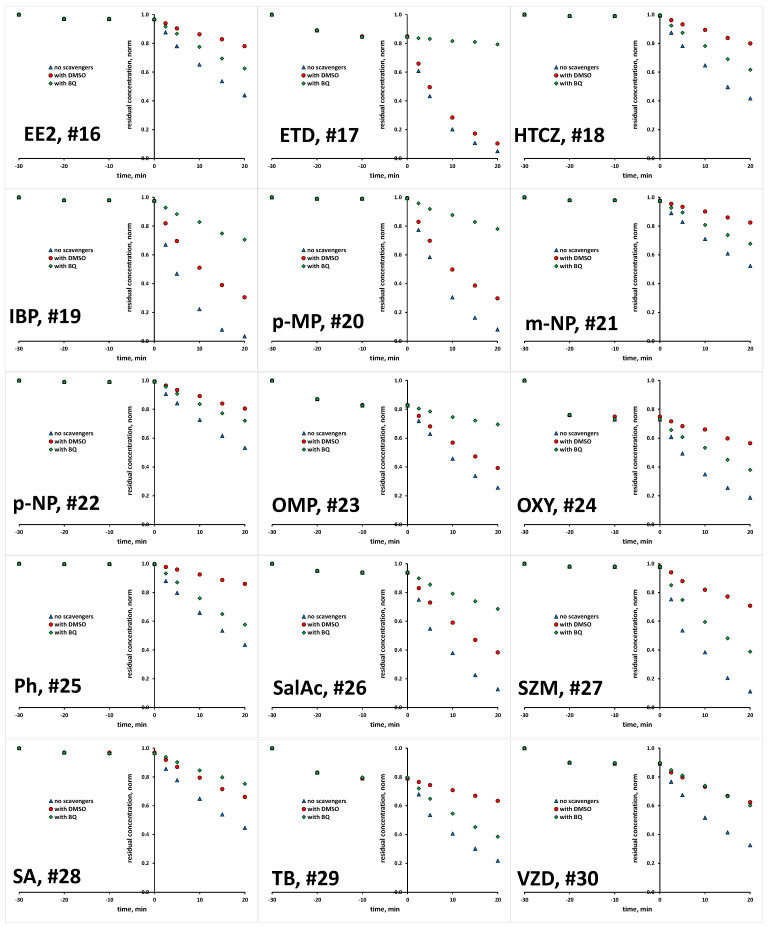
Degradation kinetics of studied organics (#16–30, Table 1) without and with the presence of scavengers for HO• and O_2_•^−^.

The scavenging agents used are effective due to the fact that react with targeted ROS at considerably high rates [29,30]. Taking into account their having a concentration 200 times higher than that of the studied organics in reaction mixture (10 mM >> 0.05 mM), ROS reactions with organics instead of scavenging agents are practically disabled. Prior experiments with scavenging agents, several blanks were performed to elucidate susceptibility of studied organics to be removed/degraded due to hydrolysis and photolysis under the UVA irradiation applied. It was established that none of the studied organics undergo hydrolysis to a significant degree; the removed portions were <0.1% within studied time course of 1 h, which covers both periods of treatment: initial dark and under UVA irradiation (results not shown). Similarly, no significant changes were recorded in photolysis experiments under UVA irradiation in the absence of TiO_2_ P25 photocatalyst over a 1 h course; portions of <0.2% were removed in all cases (results not shown). Hence, it can be established that bulk degradation occurs exclusively via ROS reactions. Besides these blank tests, we also tested the tendency of studied organics to be adsorbed onto TiO_2_ P25 surface, and as such to be removed from the reaction solution via the sorption process. A detailed investigation of the adsorption capacity of TiO_2_ P25 toward the studied organics, accompanied by response surface and QSA/PR modeling, was conducted in previous research [31], while we focus here only on the adsorption at applied photocatalytic process conditions (pH 7 and TiO_2_ loading of 0.8 gL^−1^). Most of the studied organics, as many as 17 compounds, showed very low removal (between 0 and 2%) by adsorption. Furthermore, low removal by adsorption (between 3 to 5%) was recorded for five compounds (EE2, DSL, DCF, DPH and SalAc), while higher removal values are recorded for the following organics: AMX and VZD (11%), ETD (15%), OMP (17%), TB (21%), OXY (27%) and BPA and CIP (31%) (Table S1, Supplementary Materials). In order to investigate the potential of these eight compounds for the direct degradation occurring at the catalyst surface by photogenerated *h*^+^ and *e*^−^, additional experiments with the addition of both ROS scavenging agents simultaneously (DMSO and BQ) were performed in order to suppress bulk reactions. After that, we have performed desorption tests to determine remained concentration adsorbed at the catalyst surface. In all cases, desorbed concentration corresponded to the values established as adsorbed at the catalyst surface during the initial dark period (within the mentioned experimental error). Accordingly, it can be concluded that direct oxidation/reduction of studied organics is not favorable in the studied time course, and that the majority of degradation occurs in the bulk. Hence, we were able to compare the degradation extents in tests with scavenging agents DMSO and BQ to those without, deducting the adsorbed amount from the overall concentration of targeted compound. Accordingly, we established the portion in overall degradation extent of each organic which pertained to photocatalytic degradation meditated by HO• and O_2_•^−^. The results for all 30 organics studied are presented in Figure 1 and Figure 2, and summarized in Table S1.

These relative values were then brought into correlation by creating the *K* coefficient, which denotes ratio of organics degraded by HO• ($M_{\mathrm{HO}•}$) vs. that driven by O_2_•^−^ ($M_{O_{2}{•}^{-}}$) (Equation (6)).

(6) $$K=\frac{M_{\mathrm{HO}•}}{M_{O_{2}{•}_{}^{-}}}$$

The calculated values for the *K* coefficient are provided in Table 1. In this manner, the bulk degradation mechanism can be presented by a single value, enabling easier QSA/PR modeling to establish the structural characteristics of organic pollutants that are more susceptible to HO• degradation (*K* >> 1) compared to those undergoing preferable reduction reactions via O_2_•^−^ (*K* << 1).

### 2.2. Modeling of Degradation Mechanisms over K Coefficient Using QSA/PR

The methodology applied in the correlation of the calculated *K* coefficient with the structural features of 30 studied organic compounds reflected in the calculated descriptors was well established in our previous works [22,23,31]. Hence, the set of 30 organic compounds was firstly divided into training (25 compounds) and test (five compounds) sets (Table 1). QSA/PR modeling was then applied on the training set in a step-wise fashion. Hence, models with one, two, three, four, and eventually five variables (i.e., descriptors) were developed, aiming at the highest possible accuracy (based on *R*^2^ value), simultaneously maintaining the linearity of computed models by employed QUIK rule; descriptors involved in a model cannot possess *R*_ij_ ≥ 0.6, otherwise, model is discarded. It should be noted that models with more than five variables were not considered due to the “*rule of thumb*”, which defines that a ratio higher than 1:5 between the number of variables (i.e., descriptors) in the model vs. number of compounds in the set used for modeling (i.e., training set) is not desirable [23,32]. Due to the fact that the ratio of the maximum and minimum value of *K* coefficients calculated for the selected organics was very high (~158), we tested several transformation functions (e.g., square root, log, ln, power of base 10, power of base e, etc.) to improve the modeling results. Namely, such transformations yield a narrowing of the range of responses, which usually leads to the improvement of correlation results obtained by modeling actions [31,33]. Based on the highest accuracy during preliminary modeling actions with each of applied transformation for *K*, we selected the $\frac{1}{\sqrt{\left(K+1\right)}}$ transformation and kept it in the further modeling. The benefit of using selected transformation is two-fold: besides having the highest accuracy due to suitable narrowing of responses range, it is not possible that model would predict the *K* coefficient to have a negative value, which is not practical and does not have a physical meaning.

The comparison of the performance of the one-, two-, three-, four- and five-variable QSA/PR models, selected as the best for compounds in the training and then applied on the test sets, was performed taking into account the values of statistical parameters (*R*^2^, *Q*^2^, *F*, *p*, *s*, *S*_PRESS_); the comparative values are summarized in Table S2 (Supplementary Materials).

**Figure 3:**
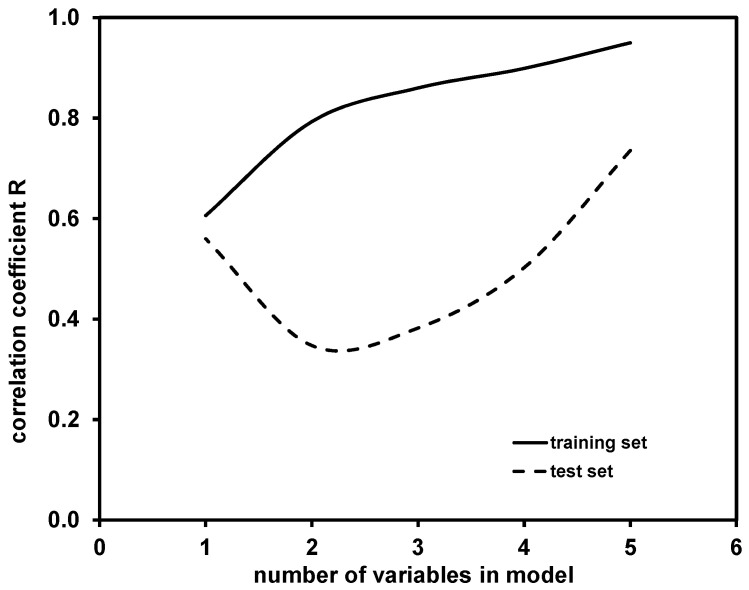
Comparison of correlation coefficients *R* obtained for training and test set by one- to five-variable models for modeling of *K* coefficient.

The main selection criterion, determining the model accuracy, was correlation coefficient of regression (*R*), for which a comparison of hte values obtained for the training and test sets is presented in Figure 3.

As can be observed, *R* values obtained for models in the training set increased with the addition of new variables into the model. A similar effect (with the exception of the one-variable model) can be observed for *R* values obtained for test set, yielding the highest accuracy in the case of five-variable model. It should be noted that higher-dimensional models, i.e., with more than five variables, might provide better predictability; however, such cases were not tested due to the above-mentioned “*rule of thumb*”. Hence, the five-variable model was selected as the best model among those calculated. That model was further validated using the Leave-Many-Out (LMO) technique [34] and the “Y-scrambling” test [35]. Graphical representations for these two validation tests are presented in Figure S1 (Supplementary Materials). It can be seen that the *Q*^2^_LMO_ values, which are not widely scattered, are rather close to the *Q*^2^_LOO_ value of the selected five-variable models and *Q*^2^_LMO_, indicating the validity of the selected QSA/PR Model (supplement, Supplementary Materials). The results of the “Y-scrambling” test further support the validity of the selected five-variable model; the *R*^2^ and *Q*^2^_LOO_ values obtained for the five-variable model are significantly higher than the values calculated for *R*^2^_Y-SCRAMBLING_ and *Q*^2^_Y-SCRAMBLING_ (supplement, Supplementary Materials). Fitting and internal and external validation criteria values for the selected five-variable model are provided in Table S3 (Supplementary Materials).

**Figure 4:**
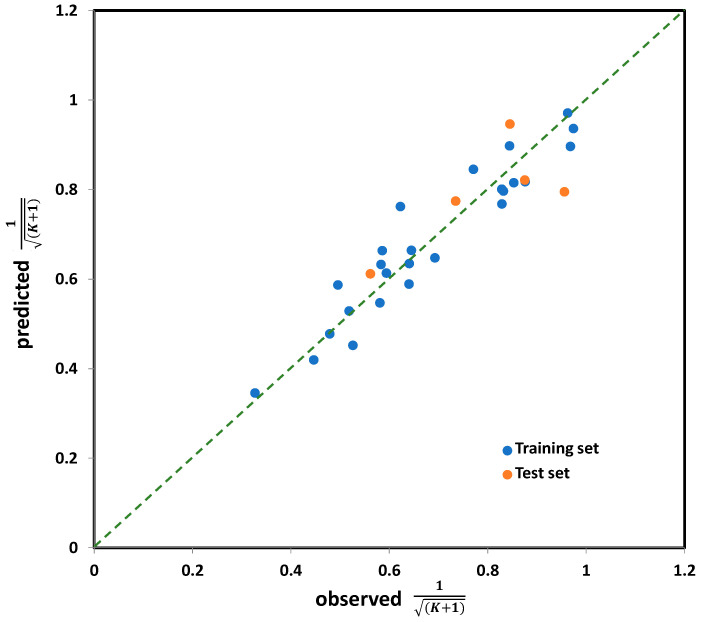
The observed vs. predicted values for *K* coefficient (in transformed $\frac{1}{\sqrt{\left(K+1\right)}}$ form), for the entire set (30 compounds) calculated by five-variable model, selected as the best one among all tested.

The performance of the selected five-variable model, when applied on the entire set (i.e., all 30 organic compounds studied), is shown in Figure 4, while the model equation is presented below (7), along with the values of corresponding statistical parameters determining its accuracy and significance.

(7) $$\begin{array}{l} Y(\frac{1}{\sqrt{\left(K+1\right)}})=-0.327\;\left(\pm 0.086\right)\times MATS4v-0.245\;\left(\pm 0.086\right)\times Mor10u\\ +0.165\;\left(\pm 0.061\right)\times CATS2D\_01\_DN-0.176\;\left(\pm 0.076\right)\times B04\left[C-Cl\right]\\ +0.151\;\left(\pm 0.067\right)\times B08\left[C-O\right]+0.533\;\left(\pm 0.053\right) \end{array}$$

(*n* = 30; *R*^2^ = 0.876; *s* = 0.069; *F* = 33.665; *p* < 0.0001; *Q*^2^ = 0.816; *S*_PRESS_ = 0.084; *S*_DEP_ = 0.076)

**Figure 5:**
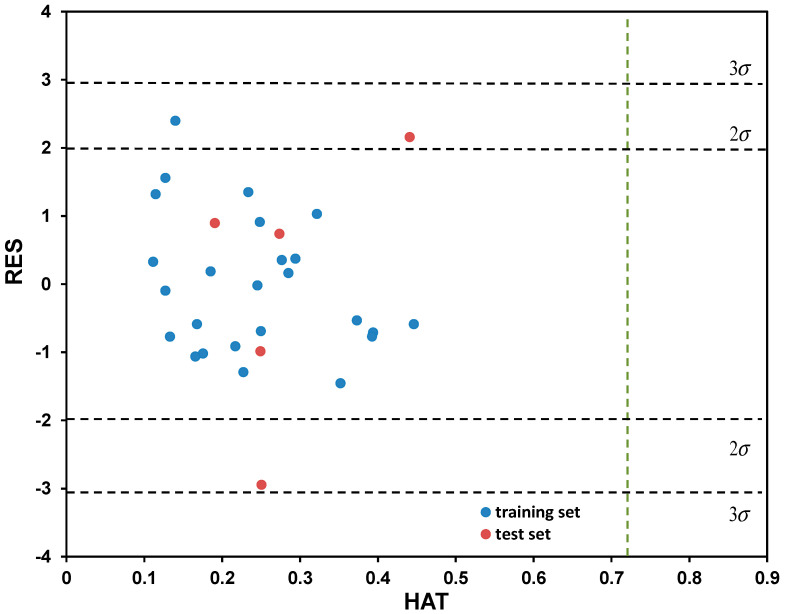
Determination of applicability domain (AD) for the five-variable model, selected as the best among tested, through Williams plot.

Based on the descriptive statistical data of the coefficients included in Model (7) that are summarized in Table S4 (Supplementary Materials), it can be concluded that all model terms are significant (i.e., all possess *p*_T_ < 0.05). The correlation matrix confirming model linearity (descriptor pairs has *R*_ij_ < 0.6) is provided in Table S5 (Supplementary Materials). As can be observed from Figure 4, the points or point clusters are placed rather close to the regression diagonal line, indicating on the high accuracy of selected five-variable Model (7). The applicability domain test of selected five-variable model was assessed employing a Williams plot (Figure 5). Leveraging such an approach enables detection of both highly structurally influential chemicals and response outliers. Hence, the limit on the *x* axis (*h*_ii_), which is calculated as h_ii_ = 3(*m* + 1)/*n*, where *m* and *n* stands for number of variables in the model and number of compounds in the training set, respectively, determines the structurally influential chemicals (based on HAT values). The limits on the *y* axis are set at ±3.0*σ*, and determine the response outliers (based on standardized RES values); these can also be associated with potential experimental errors [36]. As can be observed from Figure 5, there are no response outliers, which speaks in favor of high model predictivity, validity and accuracy. Based on the fact that there are no structurally influenced compounds (i.e., X outliers), it can be concluded that model is robust regarding the diversity of molecular structures of organic compounds.

The names, short descriptions and pertaining classes of descriptors included in the five-variable Model (7) are provided in Table 2. As can be observed, the included descriptors belong to following classes: 2D autocorrelations, 3D-MoRSE, CATS2D and 2D Atom Pairs. The first three mentioned classes include descriptors calculated by rather complex schemes, while the latter represent the occurrence of exact atom pairs at certain topological distances in the molecules. Descriptors pertaining to 2D-autocorrelations are calculated based on molecular graphs and specific algorithms such as the Broto-Moreau (AST), Geary (GATS) and Moran (MATS). Accordingly, the descriptors are denoted by the algorithm abbreviation, along with numerical properties assigned to atoms (the so-called “lag”) and the abbreviation of specific weighting scheme (*m* (relative atomic mass), *p* (polarizability), *e* (Sanderson electronegativity), *v* (Van der Waals volume), *i* (ionization potential) and *s* (I-state; electrotopological states)) [37]. Descriptors pertaining to 3D-MoRSE class (3D Molecule Representation of Structures based on Electron diffraction) are calculated by summing atom weights viewed by different angular scattering function and are denoted with the abbreviation Mor, the number for the signal, ranging from 1 to 32, and the abbreviation of the specific weighting scheme (*m*, *p*, *e*, *v*, *i* and *s*) [37]. The CATS2D class includes topological pharmacophore descriptors that are based on auto- and cross-correlation of five different pharmacophoric atom types: H-bond donor (D), H-bond acceptor (A), positively charged (P), negatively charged (N), and lipophilic (L) [38,39]. Hence, any atom in the molecule can be assigned to none, one, or two of the mentioned types, yielding 15 combinations for atom pairs. Since CATS2D descriptors are computed with the topological distance (i.e., lag) ranging from 0 to 9, overall, 150 frequencies are possible.

### 2.3. Structural Features Determining Photocatalytic Degradation Mechanisms Occurring in the Bulk

Taking into account the values of indexes of descriptors included in Model (7), the contribution of molecular features preferring degradation via HO• or O_2_•^−^ can be established. The highest contribution to the response was showed by **MATS4v**, and due to the negative index of its coefficient, this contribution is antagonistic. However, it should be noted that we used the transformed value of *K* coefficient in a form $\frac{1}{\sqrt{\left(K+1\right)}}$; thus, the lower the value of transformed *K*, the higher the original *K* value. Accordingly, what has an antagonistic effect on transformed *K* will have a synergistic effect on original *K*, meaning that the higher the *K* coefficient, the higher the portion of organic compound degraded by HO• and vice versa for O_2_•^−^. The other four included descriptors have lower absolute values of coefficients: approximately 25% (**Mor10u**), 46% (**B04[C − Cl]**), 50% (**CATS2D_01_DN**) and 54% (**B08[C − O]**). **Mor10u** and **B04[C − Cl]** have negative indexes of coefficients, having antagonistic effects on transformed *K*, but synergistic on original *K* values (i.e., denoting structural features that promote degradation via HO•). On the other hand, indexes of **CATS2D_01_DN** and **B08[C − O]** coefficients are positive, contributing eventually negatively to original *K* values (i.e., denoting structural characteristics more susceptible to degradation via reductive reaction by O_2_•^−^). Although several descriptors were obtained using the rather complex calculations, their weighting schemes may indicate the structural features more clearly. However, the descriptor **Mor10u** is unweighted. On the other hand, v, as the weighting scheme in the 2D-autocorrelation descriptor **MATS4v**, indicates the importance of Van der Waals volume, also called molecular volume and denoting the volume “occupied” by a molecule, as a general structural characteristic of a molecule attacked more preferably by either HO• or O_2_•^−^. Thus, it can be concluded that the size matters. The other three included descriptors provide more straightforward correlation of particular structural characteristics promoting the HO• driven degradation over that by O_2_•^−^. Hence, **CATS2D_01_DN** denotes a preferred topological distance (one bond) between H-bond donor and negative centers. This descriptor is characteristic for several compounds in the studied set of organics and amounts to either 1 (possessing this descriptor; compounds possessing carboxylic group (-COOH)) or 0 (compounds without this feature). Since **CATS2D_01_DN** has a positive index in Model (2), negatively contributing to the *K* value, compounds with carboxylic group are preferably degraded by O_2_•^−^. Although Chen et al. [19] emphasized the importance of carboxylic group moiety in photocatalytic degradation, a deeper correlation with the occurring mechanism and undergoing pathway was not provided. Actually, they assumed that this moiety was important within the adsorption step, which would then lead to direct degradation at the catalyst surface. However, it should be emphasized that we investigated only the indirect degradation mechanism occurring in the bulk. Furthermore, Xiao et al. [40] clearly demonstrated the role of reductive ROS in the degradation of –COOH-containing compounds; when the UV/Ag-TiO_2_ system was purged by N_2_, which diminished the dissolved O_2_ and consequently the formation of O_2_•^−^, their degradation was greatly inhibited. Hence, our results, obtained with a set of chemicals with more diversified structural differences, clearly revealed that this moiety would be favorably degraded by reduction reactions, supporting findings presented in [40]. The same influence on *K* is possessed by **B08[C − O]**, which corresponds to the presence/absence of the C–O bond at a topological distance of 8. This structural feature is characteristic for most of the CECs in the studied set, while smaller single-benzene-ring compounds are discarded due to the maximal number of C atoms either in molecule in general or C atom at too far distance from O atom. Hence, compounds possessing O as a heteroatom, either in –COOH, -O-, or –OH moieties, at this topological distance from the C atom represent preferable structural features promoting reductive degradation via O_2_•^−^. As Model (7) possesses a negative index, the descriptor **B04[C − Cl]**, which denotes the presence/absence of the C–Cl bond at a topological distance of 4, represents a structural feature preferring HO• oxidative degradation over reduction via O_2_•^−^. It should be noted that this structural feature is correlated with most of the studied organics that possess Cl as a heteroatom. The exception is AZN; although it possesses Cl, it is not counted within the **B04[C − Cl]** descriptor because the topological distance between the C and Cl atoms is lower than 4. The importance of this structural feature for favoring HO• reactions can be correlated with known degradation pathways upon attack by HO•. Namely, there are three main pathways: (i) H-abstraction (which is followed by subsequent hydroxylation); (ii) single electron transfer (SEC); and (iii) radical addition (RA) [21]. It has been established that H-abstraction is the most preferable pathway [23,26]. Hence, in a case when halide atom (e.g., Cl) is bonded to the aromatic ring, the H-abstraction pathway would comprehend the breaking of the next C–H, followed by hydroxylation at the same position in the structure. This sequence would be repeated up until ring saturation, which is then followed by its cleavage and consequently formation of open-ring, aliphatic by-products. This action is preferable to Cl atom release due to reduction reaction, which is actually mediated by O_2_•^−^ [41,42,43].

## 3. Materials and Methods

### 3.1. Chemicals

The 30 selected compounds, including 19 CECs (pharmaceuticals, pesticides, and plasticizers) and 11 common single-benzene ring aromatics, along with their names, abbreviations, CAS number, and molecular formulas, are provided in Table 1, while their structures are presented in Figure S2 (Supplementary Materials). As mentioned, the studied set includes, besides CECs, single-benzene-ring aromatics that are often used as coupling compounds for CECs or are determined as their degradation by-products. Table 1 also summarizes the calculated values of *K* coefficient (in both original and transformed values), which presents the ratio of their degradation extents in a bulk phase by HO• and O_2_•^−^; $M_{\mathrm{HO}•}$/$M_{O_{2}{•}^{-}}$. All studied organics were purchased from either Sigma Aldrich, Saint Louis, MO, or Acros Chemicals, New Jersey, NJ (both USA). The following chemicals were also used in the study either as (i) constituents of mobile phases in HPLC analysis (formic acid (HPLC grade, Sigma Aldrich, Saint Louis, MO, USA), oxalic acid (p.a., Sigma Aldrich, Saint Louis, MO, USA), methanol and acetonitrile (both HPLC grade, J.T. Baker, Phillipsburg, NJ, USA)); (ii) scavengers for HO• and O_2_•^−^ mediated bulk reactions (dimethyl sulfoxide ((CH_3_)_2_SO, DMSO, 99.9%, Sigma Aldrich, Saint Louis, MO, USA), and 1,4-benzoquinone (C_6_H_4_O_2_, BQ, 98%, Fluka, Buchs, Switzerland), respectively); or (iii) for pH adjustments (sulfuric acid (H_2_SO_4_) and sodium hydroxide (NaOH) (both p.a., Kemika, Zagreb, Croatia)). All aqueous solutions were prepared using MilliQ-water, obtained by Direct-Q3 UV (Merck Millipore, Darmstadt, Germany) ultrapure water system. The most commonly studied photocatalytic material, Aeroxide TiO_2_ P25 (Evonik, Essen, Germany), was used for UVA-driven treatment of studied organics in the presence and absence of above-mentioned ROS scavengers (DMSO and BQ).

### 3.2. Experimental Procedure

Model solutions of selected organic compounds (Table 1) with initial concentration of 0.05 mM were treated in a borosilicate-glass cylinder batch photoreactor (V = 0.08 L) with water-jacket cooling, ensuring constant temperature of reaction solution (T = 25.0 ± 0.2 °C). A light source (Pen-ray, UVP, Cambridge, UK) emitting monochromatic irradiation at 365 nm (P_0_ = 96 µW/cm^2^) was placed vertically in the middle of the reactor (*L* = 1 cm) in the quartz cuvette. The reactor was equipped with the magnetic stirrer to provide effective mixing of the reaction solution. The experiments were conducted respecting following procedure: (i) model solutions of selected organics were placed into the reactor; (ii) the appropriate amount of TiO_2_ P25 photocatalyst was added (0.8 g L^−1^); and (iii) pH was adjusted to 7 using H_2_SO_4_ or NaOH. (iv) The solution was then stirred in a dark for 30 min (based on the preliminary tests) to establish adsorption equilibrium; and (v) afterwards, the warmed-up UVA lamp was inserted in quartz cuvette and the treatment started. For each of the selected organics, three types of photocatalytic experiments were performed: (i) without scavenging agents; (ii) with DMSO (10 mM); and (iii) with BQ (10 mM) presence to suppress bulk degradation mediated by HO• and O_2_•^−^, respectively. The duration of experiments was 50 min (30 min dark period and 20 min under UVA illumination). During the experiments, 500 μL aliquots were periodically taken at −20, −10 (during dark period), 0 (starting of irradiation), 2.5, 5, 10, 15, and 20 min, filtered using Chromafil XTRA RC (25 mm, 0.45 μm, Macherey Nagel, Duren, Germany), quenched with MeOH and submitted to HPLC analysis. The experiments were conducted in quintuplicates and average values were reported; the reproducibility of experiments calculated based on HPLC measurements was 97.3%.

### 3.3. Analytical Procedure

Changes in the concentration of studied organic compounds in aqueous phase were monitored by high-performance liquid chromatography (HPLC, LC20, Shimadzu, Kyoto, Japan) equipped with UV-DAD detector (SPD-M20A, Shimadzu, Japan), two pumps and degassing unit. The injection volume was 50 μL with mobile phase flow set at 0.5 mL min^−1^. The composition of mobile phase and columns applied for the detection varied depending on the organic compound analyzed; details of HPLC analysis (including detection wavelengths) are summarized in Table S6 (Supplementary Materials). A Handylab pH/LF portable pH meter (Schott Instruments GmbH, Mainz, Germany) was used for pH adjustment monitoring.

### 3.4. Computational Part

The set of 30 organic compounds (along with calculated values of *K* coefficient) was divided into a training set (25 compounds) and a test set (5 compounds) (Table 1), respecting the same intervals of chosen responses and that extreme values are present only in a training set.

Molecular structures of organic compounds studied were built using GaussView 6.0 software (Gaussian, Inc., Wallingford, USA). The built molecular structures were then optimized using chemical density functional theory (DFT) methods (B3LYP/6-311++G(d,p)), employing Gaussian16 (Gaussian Inc., Wallingford, CT, USA) [44,45]. During this modeling action, several empirical quantum chemical parameters—(i) dipole moment (total μ, as well as its X, Y, and Z components), (ii) energy of the highest (*E*_HOMO_) and (iii) the lowest occupied molecular orbital (*E*_LUMO_) and (iv) the gap between (HLG), (v) final heat of formation (Δ*H*_f_), and (vi) ionization potential—were calculated and later used as theoretical descriptors. The majority of the molecular descriptors were calculated employing DRAGON 6.0 software (Milano Chemometrics and QSAR Research Group, TALETE, Milano, Italy) using optimized molecular structures of studied organic compounds by DFT. In this manner, 3129 molecular descriptors were obtained which captured relevant structural features of studied organic compounds, thus describing their chemical diversities.

The correlation between QSA/PR responses (*K* coefficients) and descriptors calculated by DRAGON and DFT was obtained employing the combined approach which included variable selection Genetic Algorithm (GA) and Multiple Linear Regression Analysis (MLRA) embedded within QSARINS 2.2.4 (QSAR Group, University of Insubria, Italy) [46,47,48]. In this manner, the most influential descriptors were selected within built 1–5-variable models (i.e., including 1 to 5 descriptors). The GA variable selection technique started with following parameters: 200 random models, the generation size of 2000 iterations, and the mutation probability specified as 20%.

Before the above-mentioned modeling actions using QSARINS 2.2.4 software, the descriptor matrix was screened for highly intercorrelated and redundant descriptors. Accordingly, descriptor pairs with *R* values ≥ 0.99 were removed (overall, 496 descriptors). In this manner, the overall number of descriptors in the matrix was reduced to 2642, including 2633 Dragon calculated and 9 computed by DFT. In order to enable the comparison of contributions of descriptors involved in generated QSA/PR models to the end-point responses (i.e., *K* coefficients), descriptor matrix was then normalized. During GA and MLRA modeling actions, the filtering rules were employed to discard models with either highly correlated descriptors (*R*_ij_ > 0.6) or those with the inadequate significance (*p*_M_ or *p*_T_ ≥ 0.05); QUIK rule was applied prior modeling, CI rule was activated after models were built. The validation and verification of models was based on common statistical parameters (Table 3), as well as Leave Many Out (LMO) and “Y-scrambling” tests. The applicability domain (AD) of the selected models was estimated using a Williams plot [49], where the response outliers and structurally influential compounds could be straightforwardly detected.

## 4. Conclusions

A QSA/PR model was developed aiming at describing the structural diversity of contaminates of emerging concern (CECs) during photocatalytic treatment susceptible to the occurrence of oxidative and reductive mechanisms driven by HO• and O_2_•^−^, respectively. First, we determined the degradation rates of the targeted organics in the absence and presence of common scavengers for HO• and O_2_•^−^ during their photocatalytic treatment. The obtained values were then brought into correlation via the *K* coefficient, denoting the ratio of organics degraded by two occurring mechanisms; *K* >> 1 compounds are more susceptible to HO• degradation, while *K* << 1 compounds prefer reduction reactions driven by O_2_•^−^. The values of *K* coefficient were then used as responses in quantitative structure–property relationship (QSA/PR) modeling. The QSA/PR modeling included validation over internal validation parameters, as well as by Leave-Many-Out (LMO) and “Y-scrambling” tests, which showed that the selected model was statistically significant. Furthermore, the applicability domain of the model selected as the best was defined by the leverage approach using a Williams plot to detect the highly structurally influential chemicals and response outliers.

The results of the QSA/PR modeling revealed that structural features such as the size of the molecules, represented by the **MATS4v** descriptor, influence the degradation rate in general. Furthermore, the presence/absence of particular molecular fragments such as C − O (particularly in a form of carboxyl group), represented by **CATS2D_01_DN** and **B08[C − O]** descriptors, and C − Cl bonds, represented by the **B04[C − Cl]** descriptor, dictate the preferable degradation pathway of CECs by photocatalytic degradation; the latter favors HO• driven reaction, while the former favors the reductive pathway. The developed QSA/PR models can be considered robust predictive tools for evaluating distribution between degradation mechanisms occurring in photocatalytic treatment, and guidance for tailoring photocatalyst to favor oxidative or reductive reactions, depending on the structure of targeting pollutants.

## Figures and Tables

**Table 1:** List of 30 selected organics used in a study, along with original and transformed values of *K* coefficient, with included splitting into training and ***test*** set for QSA/PR modeling.

#	Compound	Abbreviation	CAS	Molecular Formula	*K*	$\frac{1}{\sqrt{\left(K+1\right)}}$
1	Alachlor	ALC	15972-60-8	C_14_H_20_ClNO_2_	2.722	0.518
2	o-Aminobenzoic acid	o-aminoBenzAc	118-92-3	C_7_H_7_NO_2_	0.079	0.963
***3***	***Amoxicillin***	***AMX***	***26787-78-0***	***C_16_H_19_N_3_O_5_S***	***0.095***	***0.956***
4	Atrazine	AZN	1912-24-9	C_8_H_14_ClN_5_	4.017	0.446
5	Benzoic acid	BenzAc	65-85-0	C_6_H_5_COOH	0.374	0.853
6	Bisphenol A	BPA	80-05-7	C_15_H_16_O_2_	1.580	0.623
***7***	***Ciprofloxacin***	***CIP***	***85721-33-1***	***C₁₇H₁₈FN₃O₃***	***0.852***	***0.735***
8	Desloratadine	DSL	100643-71-8	C_19_H_19_ClN_2_	1.442	0.640
9	Desvenlafaxine	DVF	93413-62-8	C_16_H_25_NO_2_	0.445	0.832
10	2,4-Dichlorophenol	DCP	120-83-2	C_6_H_4_Cl_2_O	3.358	0.479
11	Diclofenac	DCF	15307-79-6	C_14_H_10_Cl_2_NNaO_2_	0.457	0.829
**12**	**1,4-Dimethoxybenzene**	**1,4-DMB**	**150-78-7**	***C_6_H_4_(OCH_3_)_2_***	**0.400**	**0.845**
13	2,6-Dimethoxyphenol	2,6-DMP	91-10-1	(CH_3_O)_2_C_6_H_3_OH	0.053	0.974
14	Diuron	DIU	330-54-1	C_9_H_10_Cl_2_N_2_O	8.358	0.327
15	Donepezil HCl	DPH	120011-70-3	C_24_H_30_ClNO_3_	1.404	0.645
16	17α-Ethynylestradiol	EE2	57-63-6	C_20_H_24_O_2_	1.833	0.594
17	Etodolac	ETD	41340-25-4	C_17_H_21_NO_3_	0.067	0.968
18	Hydrochlorothiazide	HCTZ	58-93-5	C_7_H_8_ClN_3_O_4_S_2_	1.940	0.583
19	Ibuprofene	IBP	15687-27-1	C_13_H_18_O_2_	0.402	0.844
***20***	***p-Methoxyphenol***	***p-MP***	***150-76-5***	***CH_3_OC_6_H_4_OH***	***0.305***	***0.875***
21	m-Nitrophenol	m-NP	554-84-7	O_2_NC_6_H_4_OH	1.965	0.581
22	p-Nitrophenol	p-NP	100-02-7	O_2_NC_6_H_4_OH	1.437	0.641
23	Omeprazole HCl	OMP	73590-58-6	C_17_H_20_ClN_3_O_3_S	0.302	0.876
24	Oxytetracycline	OXY	79-57-2	C_22_H_24_N_2_O_9_	1.916	0.586
25	Phenol	Ph	108-95-2	C_6_H_5_OH	3.069	0.496
26	Salicylic acid	SalAc	69-72-7	C_7_H_6_O_3_	0.455	0.829
***27***	***Simazine***	***SZM***	***122-34-9***	***C_7_H_12_ClN_5_***	***2.171***	***0.562***
28	Sulfanilic acid	SA	121-57-3	C_6_H_7_NO_3_S	0.682	0.771
29	Tobramycin	TB	32986-56-4	C_18_H_37_N_5_O_9_	2.614	0.526
30	Vilazodone HCl	VZD	163521-08-2	C_26_H_27_N_5_O_2_	1.084	0.693

**Table 2:** Definitions of descriptors included in the five-variable model for prediction of *K* coefficient.

Descriptor Name	Descriptor Definition	Descriptor Type
**MATS4v**	Moran autocorrelation of lag 4 weighted by van der Waals volume	2D autocorrelations
**Mor10u**	signal 10/unweighted	3D-MoRSE
**CATS2D_01_DN**	CATS2D Donor-Negative at lag 01	CATS 2D
**B04[C − Cl]**	Presence/absence of C − Cl at topological distance 4	2D Atom Pairs
**B08[C − O]**	Presence/absence of C O at topological distance 8	2D Atom Pairs

**Table 3:** Definitions of descriptors included in five-variable model for the prediction of the *K* coefficient.

#	Symbol	Definition
1	*R*	the correlation coefficient of regression
2	*R* ^2^	the model explained variance
3	*Q* ^2^	the leave-one-out cross-validation coefficient
4	*F*	F-ratio between variances of observed and calculated values
5	*p*	probability value for calculated *F*
6	*s*	standard error
7	*S*_PRESS_	standard error of the predictive residue of sum of squares

## Data Availability

The datasets collected and analyzed within this work are available from the corresponding authors upon written request.

## Supplementary Materials

The following supporting information can be downloaded at: https://www.mdpi.com/article/10.3390/molecules28062443/s1, Figure S1: Scatter plots of LMO and Y-scrambling model compared to the 5-variable QSA/PR model; Figure S2: Molecular structures of studied contaminants of emerging concern and single-benzene ring compounds; Table S1: Experimental results on the removal and degradation of studied organics by UVA/TiO_2_ process (pH_0_ 7 and TiO_2_ loading of 0.8 gL^−1^); Table S2: Statistical evaluation of QSA/PR models for training set (25 compounds) and test set (5 compounds); Table S3: Values of fitting, internal and external validation criteria of selected best 5-variable model; Table S4: Descriptive statistical data included in the best 5-variable model; Table S5: Correlation matrix of descriptors included in best 5 variable model for entire set of compounds (cross-correlation *R*_ij_ < 0.6); Table S6: Composition of mobile phases and detection details for HPLC analysis of 30 studied organics.

## Sample Availability

Samples of the compounds are not available from the authors.
