# Supplementary material for: Structural Features Promoting Photocatalytic Degradation of Contaminants of Emerging Concern: Insights into Degradation Mechanism Employing QSA/PR Modeling
Source: Molecules. 2023 Mar 7;28(6):2443. doi: 10.3390/molecules28062443 (PMC10057466; doi:10.3390/molecules28062443)

Supplementary material

Which Factors Influence the Immensely Fluctuating CRT Implantation Rates in Europe? A Mixed Methods Approach Using Qualitative Content Analysis Based on Expert Interviews

Christoph Edlinger [1,2,3,*], Marwin Bannehr [1,2], Christian Georgi [1,2], David Reiners [1,2], Michael Lichtenauer [3], Anja Haase-Fielitz [1,2] and Christian Butter [1,2]

[1] Department of Cardiology, Heart Center Brandenburg, 16321 Berlin, Germany

[2] Faculty of Health Sciences Brandenburg, Brandenburg Medical School (MHB) "Theodor Fontane", 16816 Neuruppin, Germany

[3] Clinic of Internal Medicine II, Department of Cardiology, Paracelsus Medical University of Salzburg, 5020 Salzburg, Austria

* Correspondence: christophroland.edlinger@immanuelalbertinen.de or christoph.edlinger@mhb-fontane.de

Table S1. Experimental results on the removal and degradation of studied organics by UVA/TiO_2 process (pH_0 7 and TiO_2 loading of 0.8 gL^{-1})

#	Abbreviation	Overall removal, %	Removal due to adsorption, %	Removal due to degradation, %	k_{obs}, s^{-1} ($\times10^{-2}$)	Relative removal mediated by HO•	$k(HO•)_{obs}$, s^{-1} ($\times10^{-2}$)	Relative removal mediated by $O_2•^-$	$k(O_2•^-)_{obs}$, s^{-1} ($\times10^{-2}$)	*K*
1	ALC	90.68	1.55	89.13	11.98	72.60	5.28	26.67	1.41	2.722
2	*o*-aminoBenzAc	77.50	1.99	75.51	7.63	7.31	0.03	92.42	6.34	0.079
3	AMX	69.05	11.00	58.05	5.35	8.58	0.31	90.57	4.44	0.095
4	AZN	45.72	1.19	44.35	2.90	79.74	2.24	19.85	0.47	4.017
5	BenzAc	80.86	0.59	80.27	8.28	27.16	1.23	72.56	4.40	0.374
6	BPA	67.17	31.54	35.63	3.79	60.87	1.92	38.52	1.14	1.580
7	CIP	98.00	30.71	67.29	17.34	30.50	1.69	68.96	5.66	0.852
8	DSL	74.89	4.89	70.00	6.81	58.91	2.95	40.85	1.67	1.442
9	DVF	70.27	0.36	69.91	5.94	30.77	1.20	69.14	3.43	0.445
10	DCP	59.05	1.79	57.26	4.41	76.76	3.03	22.86	0.74	3.358
11	DCF	78.81	5.00	73.81	7.89	31.28	1.45	68.49	3.94	0.457
12	1,4-DMB	90.89	1.73	89.16	11.99	28.51	1.49	71.25	5.22	0.400
13	2,6-DMP	99.54	0.71	98.84	15.23	5.04	0.27	94.51	13.15	0.053
14	DIU	78.67	1.99	76.68	7.52	88.60	5.84	10.60	0.45	8.358
15	DPH	63.31	5.39	57.91	4.70	78.77	3.33	21.04	0.69	1.404
16	EE2	56.08	3.21	52.87	3.96	64.56	2.19	35.21	1.07	1.833
17	ETD	94.92	15.27	79.65	13.98	6.29	0.03	93.55	10.61	0.067
18	HCTZ	58.23	0.62	57.61	4.44	65.58	2.42	33.80	1.11	1.940
19	IBP	95.86	1.80	94.16	16.40	28.67	1.68	71.26	6.04	0.402
20	*p*-MP	91.83	0.63	91.19	12.19	23.31	1.22	76.46	6.28	0.305
21	*m*-NP	47.42	2.07	45.35	3.15	65.81	1.84	33.48	0.84	1.965
22	*p*-NP	46.76	0.64	46.12	3.15	58.89	1.64	40.97	1.09	1.437
23	OMP	74.40	17.41	56.99	5.88	23.15	0.09	76.55	3.75	0.302
24	OXY	81.24	27.16	54.08	6.96	65.08	3.27	33.97	1.43	1.916
25	Ph	56.46	0.24	56.22	4.17	74.96	2.77	24.43	0.76	3.069
26	SalAc	85.26	4.20	81.06	9.73	31.22	1.61	68.58	4.57	0.455
27	SZM	88.53	1.93	86.60	10.54	68.27	4.74	31.45	1.66	2.171
28	SA	53.90	1.95	51.95	3.91	40.43	1.24	59.25	1.96	0.682
29	TB	78.28	21.00	57.28	6.51	71.91	3.72	27.51	1.12	2.614
30	VZD	67.45	10.74	56.71	5.14	51.73	1.98	47.73	1.90	1.084

Table S2. Statistical evaluation of QSA/PR models for training set (25 compounds) and test set (5 compounds)

Model	Training set							Test set			
	n	R^2	Q^2	F	*p*	*s*	S_{PRESS}	n	R^2	*F*	*s*
1-variable	25	0.367	0.274	13.372	0.0013	0.145	0.156	5	0.313	1.825	0.064
2-variable		0.629	0.546	18.639	p<0.0001	0.114	0.126		0.121	0.548	0.081
3-variable		0.740	0.637	19.911	p<0.0001	0.097	0.115		0.147	0.684	0.144
4-variable		0.808	0.731	20.971	p<0.0001	0.086	0.101		0.252	1.349	0.075
5-variable		0.902	0.841	35.004	p<0.0001	0.063	0.080		0.541	4.716	0.044

Table S3. Values of fitting, internal and external validation criteria of selected best 5-variable model

Fitting criteria		Internal validation criteria		External validation criteria	
R^2	0.902	Q^2_{loo}	0.841	R^2_{ext}	0.541
R^2_{adj}	0.876	R^2-Q^2_{loo}	0.060	Q^2F_1	0.719
R^2-R^2_{adj}	0.026	Q^2_{LMO}	0.818	Q^2F_2	0.537
LOF	0.008	$PRESS_{cv}$	0.121	Q^2F_3	0.719
$RMSE_{tr}$	0.055	$RMSE_{cv}$	0.070	$PRESS_{ext}$	0.043
MAE_{tr}	0.045	MAE_{cv}	0.059	$RMSE_{ext}$	0.093
CCC_{tr}	0.948	CCC_{cv}	0.917	MAE_{ext}	0.081
		R^2Y_{scr}	0.206	CCC_{ext}	0.714
		Q^2Y_{scr}	-0.399	R^2_{ext}	0.541

Table S4. Descriptive statistical data included in the best 5-variable model

predictor	Coef	Stdev	95% Conf	t-ratio	*p**
**Constant**	0.533	0.026	0.053	20.727	0
**MATS4v**	-0.294	0.037	0.077	-7.862	0
**Mor10u**	-0.245	0.042	0.086	-5.854	0
**CATS2D_01_DN**	0.165	0.030	0.061	5.545	0
**B04[C-Cl]**	-0.176	0.033	0.076	-4.789	0.0001
**B08[C-O]**	0.151	0.033	0.067	4.648	0.0001

**p*<0.05 is significance limit

Table S5. Correlation matrix of descriptors included in best 5 variable model for entire set of compounds (cross-correlation R_{ij}<0.6)

	MATS4v	Mor10u	CATS2D_01_DN	B04[C-Cl]	B08[C-O]
**MATS4v**	1	0.076	0.016	0.187	0.184
**Mor10u**	0.076	1	0.025	0.49	0.542
**CATS2D_01_DN**	0.016	0.025	1	0.113	0.233
**B04[C-Cl]**	0.187	0.49	0.113	1	0.269
**B08[C-O]**	0.184	0.542	0.233	0.269	1

Table S6. Composition of mobile phases and detection details for HPLC analysis of 30 studied organics

Compound	*w*(PHASE 1)	*w*(PHASE 2)	*λ*, nm	Column
o-Aminobenzoic acid	0.50 (CH_3OH)	0.50 (H_2O)	333	Machery C18 250 x 4,6mm, 4,6μm
17α-Ethynylestradiol	0.70 (CH_3OH)	0.30 (H_2O)	282	
Etodolac	0.60 (CH_3CN)	0.40 (H_2O)	219	
Omeprazole HCl	0.30 (CH_3CN)	0.70 (H_2O)	278	
Amoxicillin	0.10 (CH_3CN)	0.90 (H_2O)	273	
Benzoic acid	0.50 (CH_3CN)	0.50 (H_2O)	228	
Hydrochlorothiazide	0.50 (CH_3CN)	0.50 (H_2O)	270	
Diuron	0.60 (CH_3CN)	0.40 (H_2O)	219	
Diclofenac	0.70 (0.1% CH_2O_2)	0.30 (H_2O)	276	
Ibuprofene			219	
Salicylic acid			303	
Bisphenol A	0.60 (CH_3OH)	0.40 (0.2% CH_2O_2)	276	
Sulfanilic acid	0.10 (CH_3OH)	0.90 (0.2% CH_2O_2)	254	
Phenol	0.50 (CH_3OH)	0.50 (0.1% CH_2O_2)	270	
p-Nitrophenol			318	
p-Methoxyphenol			318	
m-Nitrophenol			318	
2,6-Dimethoxyphenol			210	
2,4-Dichlorophenol			288	
1,4-Dimethoxybenzene			288	
Alachlor	0.40 (CH_3CN)	0.60 (0.1% CH_2O_2)	276	
Atrazine			230	
Simazine			230	
Desvenlafaxine	0.30 (CH_3OH)	0.70 (0.2% CH_2O_2)	318	Shodex 4,6 mm IDx150 mm
Donepezil HCl			318	
Desloratadine			276	
Oxytetracycline	0.35 (CH_3OH)	0.65 (10mM $C_2H_2O_4$)	354	
Vilazidone HCl			273	
Tobramycin	0.40 (CH_3OH)	0.40 (H_2O)	273	
Ciprofloxacin	0.10 (CH_3CN)	0.90 (0.2% CH_2O_2)	273	Atlantis T3 5 μm, 4.6 mm X 150 mm

Figure S1. Scatter plots of LMO and Y-scrambling model compared to the 5-variable QSA/PR model

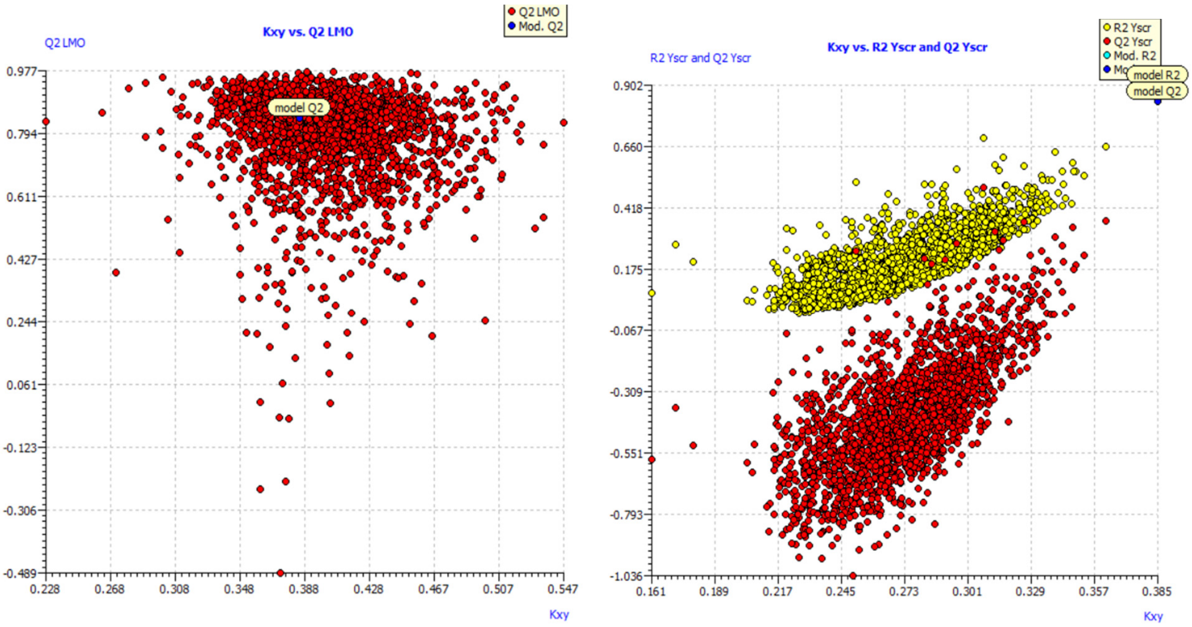

Figure S2. Molecular structures of studied contaminants of emerging concern and single-benzene ring compounds

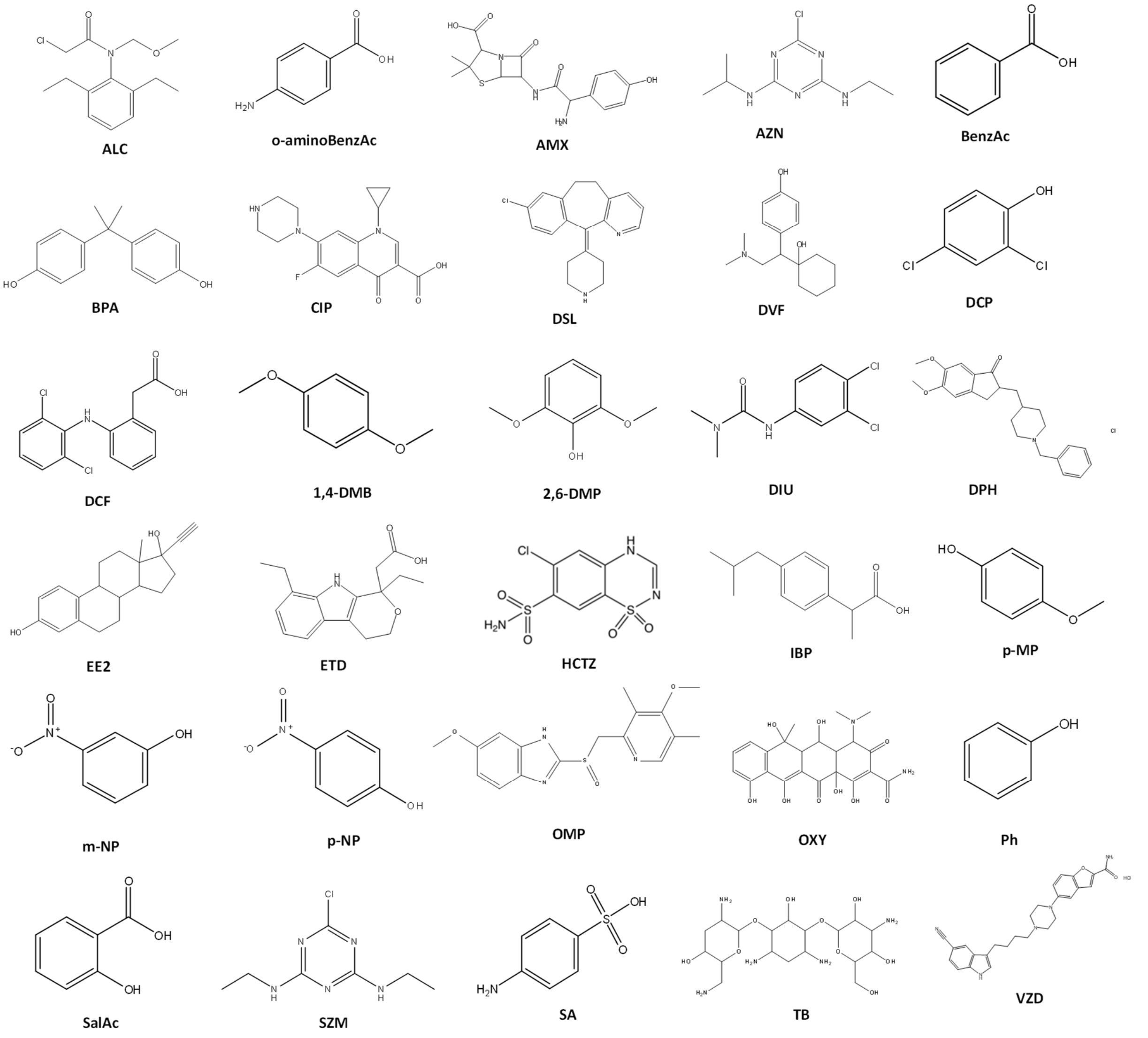

Supplement: Supplementary file 1 [file molecules-28-02443-s001.zip › molecules-2258905-supplementary.pdf]
